# Automatic Measurement of the Carotid Blood Flow for Wearable Sensors: A Pilot Study

**DOI:** 10.3390/s21175877

**Published:** 2021-08-31

**Authors:** Riccardo Matera, Stefano Ricci

**Affiliations:** Information Engineering Department (DINFO), University of Florence, 50139 Firenze, Italy; riccardo.matera@unifi.it

**Keywords:** Doppler velocity measurement, blood velocity, vector Doppler, automatic carotid segmentation, wearable sensors

## Abstract

The assessment of the velocity of blood flowing in the carotid, in modern clinical practice, represents an important exam performed both in emergency situations and as part of scheduled screenings. It is typically performed by an expert sonographer who operates a complex and costly clinical echograph. Unfortunately, in developing countries, in rural areas, and even in crowded modern cities, the access to this exam can be limited by the lack of suitable personnel and ultrasound equipment. The recent availability of low-cost, handheld devices has contributed to solving part of the problem, but a wide access to the exam is still hampered by the lack of expert sonographers. In this work, an automated procedure is presented with the hope that, in the near future, it can be integrated into a low-cost, handheld instrument that is also suitable for self-measurement, for example, as can be done today with the finger oximeter. The operator should only place the probe on the neck, transversally with respect to the common tract of the carotid. The system, in real-time, automatically locates the vessel lumen, places the sample volume, and performs an angle-corrected velocity measurement of the common carotid artery peak velocity. In this study, the method was implemented for testing on the ULA-OP 256 scanner. Experiments on flow phantoms and volunteers show a performance in sample volume placement similar to that achieved by expert operators, and an accuracy and repeatability of 3.2% and 4.5%, respectively.

## 1. Introduction

The echo-Doppler [1] investigation of the velocity of blood that flows in the carotid artery is one of the most practiced exams in current clinical activity. It is employed, for example, to investigate general cardiovascular conditions, to inspect the presence of dangerous atherosclerotic plaques [2], to monitor the hemodynamics of patients who have undergone endarterectomy or stent insertion [3], etc. The exam is typically carried out by an expert sonographer who operates a costly and complex echographic platform installed in a clinical environment. The expert operator checks the correct probe position in the live B-mode image; locates the region of interest (ROI) in the middle of the carotid lumen; places the Doppler line and the sample volume; evaluates manually the Doppler angle; finally, the operator switches the echograph’s echo-Doppler modality to obtain velocity data [1].

Unfortunately, echographic equipment is often not as available as it needs to be. This is apparent for developing countries and/or rural areas [4], but it also applies to densely populated cities in developed countries where hospitals and health-care points are often insufficient and overutilized, and where governments are striving for a more efficient use of public resources [5]. Telesonography, i.e., the use of ultrasound scanners from a remote site, could reduce the problem, but the current scanners are often not suitable for this employment [6].

Recently, application-specific integrated circuits (ASICs), which include important sections of the electronics needed in an ultrasound system, have been developed. They integrate, for example, the receiver front end and the beamformer [7], the transmitter and the piezoelectric transducers array [8], and the complete transmission–reception transceiver [9]. The availability of these devices fosters the development of compact and economical ultrasound systems [10,11,12,13,14], even based on smartphones [15], and can change the ultrasound paradigm, opening new scenarios for their employment. These apparatuses represent an important contribution, but they alone do not completely solve the problem, since, as noted before, the presence of an expert operator is still required.

The vision that promoted this work is the realization of a very simple, low-cost, handheld, automatic system for the measurement of the blood velocity peak in the common carotid artery (CCA). Ideally, it can be used in drugstores, or can even be sold in markets and employed at home, as it is today for self-medication tools that monitor blood pressure and oxygen saturation. Hard compromises are required in pursuing this target. The measurement accuracy and reliability of such a tool will never compete with those of a high-end echograph operated by an expert sonographer. Instead, this tool is expected to produce a reasonable measurement that, for example, can trigger an alarm in a patient and induce her/him to submit to a full clinical ultrasound exam at a later date. The smart device presented in [16] goes in this direction: it employs a large continuous wave (CW) sample volume to cover the carotid, but does not compensate for angle or for patient movement.

This work contributes to this vision by presenting a suitable sensor geometry and the associated ultrasound method. The proposed sensor features a relatively wide field of view that maximizes the chances for the non-expert user to locate the vessel even without the help of a B-mode display; an algorithm automatically locates the vessel lumen in the B-mode images [17], places the Doppler lines in the right position, and performs an angle-independent velocity measurement. This last step is achieved with the use of two angled linear arrays that intercept the carotid in a transversal position and perform a dual-beam vector Doppler velocity measurement [18]. The user is requested only to place the probe on the neck so that it crosses the CCA transversally and to start the procedure, which lasts a few seconds [19].

This method is targeted at being implemented in embedded tools possibly based on integrated transducers technology, such as in [8]. However, before developing a specific hardware, we adapted a ULA-OP 256 research echograph [20] to test the performance and the suitability of the proposed method.

## 2. Materials and Methods

### 2.1. Probe Geometry

**Figure 1:**
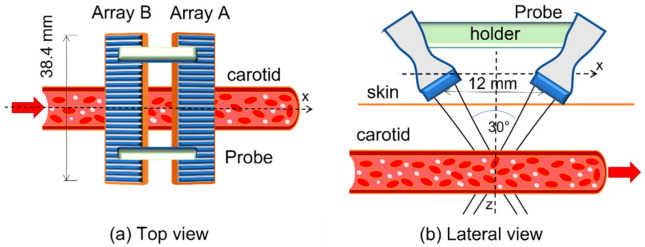
The proposed probe is composed of 2 linear arrays of 38.4 mm lateral extension, spaced by 11 mm. The arrays are tilted so that a 30° angle is present between their axes.

In this work, we employed 2 commercial linear probes L12-5D (Acoustic Life Science Co., Ltd., Shanghai, China), with 128 transducers each (see Table 1 for details). A probe holder was built to arrange the 2 arrays (Array A and Array B) in the configuration sketched in Figure 1. The arrays should be placed in a transversal position with respect to the vessel. Their depth axes form a 30° inter-angle, and this inclination allows investigation of the blood flow from 2 different directions, making it possible for automatic Doppler angle compensation through a dual-beam approach [18,21]. The distance between the 2 arrays was calculated so that their axes cross at about 23 mm of depth, which is compatible with the typical depth of the carotid artery [22]. In this work, we employed an active aperture of 28.8 mm, corresponding to 96 out of the 128 elements available in the probes.

### 2.2. Echograph Integration

The proposed method was integrated in the experimental echograph ULA-OP 256 [20]. The scanner was equipped with a special board that allowed the simultaneous connection of the 2 probes. This board hosted 2 QLC260R ITT Cannon (Irvine, CA, USA) connectors, and routed the 128 transducers of each probe in the 256 channels of the scanners so that ULA-OP256 could manage the 2 probes as in a single 256-element probe.

ULA-OP 256 was connected to MATLAB^®^ (The MathWorks, Natick, MA, USA), which, through a proprietary interface, was able to manage the scanner in a quasi-real-time modality. In this modality, MATLAB^®^, following the instruction of the script edited by the designer, sets the parameters, starts/stops the acquisition, acquires, and processes the data without the direct intervention of the user.

### 2.3. Operation Sequence

The MATLAB^®^ script that commands the scanner was developed so that the whole investigation occurs with minimal operator involvement. It is sufficient that the user, even without the help of the B-mode display, places the probes roughly over the common tract of the carotid in transversal orientation, and starts the script execution.

The investigation is divided in more phases: at the beginning, MATLAB^®^ sets the scanner for B-mode and acquires a sequence composed of 15 images from both the left and right probes. A standard line-by-line transmission (TX)–reception (RX) strategy is employed according to the parameters reported in Table 2. Channel samples are beamformed, demodulated in-phase/quadrature (IQ) data on board of the scanner, and automatically transferred in MATLAB^®^. MATLAB^®^ immediately processes the images to locate the carotid and to extract the center of its lumen, independently of the left and right B-mode sequences (see Section 2.4 for details). Successively, MATLAB^®^ switches the scanner into Doppler mode, with the parameters detailed in Table 2. Two non-steered investigation lines (one for each probe) are placed. These lines cross the carotid center according to the lateral coordinates found in the previous step. Approximately 2 s of Doppler data are acquired, with transmissions alternated between the 2 probes at pulse repetition frequency (PRF) of 16 kHz (8 kHz for each line). The relatively long temporal length was chosen to obtain a complete heart cycle under all circumstances. Raw Doppler data are coherently demodulated [23] and automatically moved and processed in MATLAB^®^, as detailed in Section 2.5. The patient is expected not to move during the investigation that, anyhow, lasts no more than 3 s.

### 2.4. Automatic Detection of the Vessel Center

The aim of the algorithm here described is to detect the lateral position of the carotid lumen when the array investigates the vessel transversally. The lumen is relatively easy to be located: it appears as a dark anechoic region surrounded by tissue with different echogenicity, which, typically, is clearer. Following the array inclination, a perfectly cylindrical vessel produces an elliptical shape, but its ellipticity is so low that the section can be safely approximated to a circular shape.

**Figure 2:**
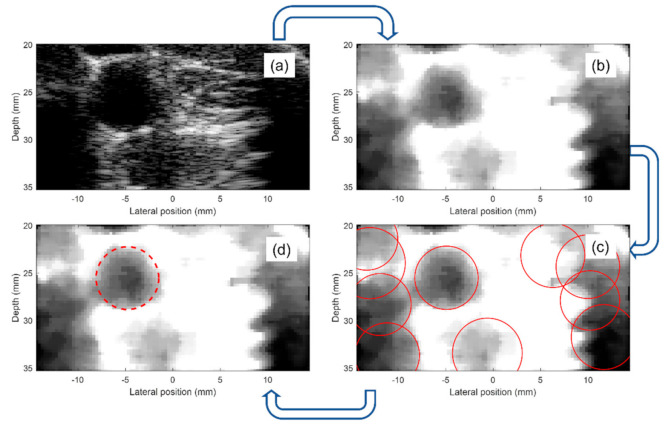
Procedure for automatic carotid segmentation. (**a**): The raw B-Mode image is acquired; (**b**): the image is pre-processed; (**c**): candidate carotids (red circles) are detected; (**a**–**c**) is repeated over 15 images to obtain the candidate matrix; (**d**): the final segmentation is selected.

In summary, the algorithm must locate a dark circle in a background of variable grey levels. We developed an algorithm based on the circular Hough transform (CHT) [24], which represents a well-established method for detecting curves and shapes in images [25]. The algorithm proceeds as follows: for each frame of the B-mode sequence (Figure 2a), the image is pre-elaborated to optimize the brightness and contrast. Then, a strong gaussian filter reduces the noise (Figure 2b). Although several thin morphological structures are destroyed in this process, the big artery lumen is emphasized. Then, the CHT detects all the dark circles whose radii are between 2.1–4 mm [26] (Figure 2c). The detected center coordinates (xi, yi) and radii (ri) are collected. For each of the dark circles that have been detected, the brightness values of their pixels are assessed. The circle showing the darkest values is elected as “candidate circle”, and its center coordinates (xci, yci) with radius (rci) are saved in a matrix of candidates. Once all the B-Mode frames sequence has been processed, the matrix holds the results obtained from each image. The most frequent (xc, yc, rc) triad occurring in the matrix is selected as the final choice (red circle in Figure 2d).

### 2.5. Flow Assessment

As detailed in the previous section, the multi-gate (512 depths) IQ Doppler data acquired from the 2 probes are moved in MATLAB^®^. The 2 depth coordinates of the vessel lumen, located in the step described in Section 2.4, are averaged and used as center of a 6 mm Doppler region of interest (ROI) where, presumably, the depth of the maximum velocity (which is not necessarily the vessel center) is located. Data from the ROI are further processed with a 100 Hz clutter filter. Data are divided in 128-sample packets with a 50% overlap, and processed through fast Fourier transform (FFT). The centroid Doppler frequency of each spectrum is extracted by a modified center-mass algorithm [27] to obtain the frequency profile along depths. The frequencies at the same depth and time, detected in the ROI from the right and left probes, $f_{dr}$ and $f_{dl}$, are triangulated. The velocities along lateral ($V_{x}$) and axial ($V_{z}$) directions (see Figure 1) are detected as in [21]

(1) $$\begin{array}{cc} V_{x}=\frac{c}{4f_{tx}}\;\frac{f_{dr}-f_{dl}}{\sin\frac{\theta }{2}},\; & V_{z}=\frac{c}{4f_{tx}}\;\frac{f_{dr}+f_{dl}}{\cos\frac{\theta }{2}} \end{array}$$

where *c* is the sound velocity, $f_{tx}$ is the transmission frequency, and θ is the inter-axes angle between the probes. The angle-corrected velocity is finally obtained

(2) $$V=\sqrt{V_{x}{}^{2}+V_{z}{}^{2}}$$

In other words, the system calculates the sequence of the angle-corrected velocity profiles in time, i.e., a profile every 64 Doppler TX pulses (128 packet size with 50% overlap). Finally, the maximum velocity detected along the profile (depth) and along time (profile temporal sequence) is selected. This single value represents the measurement output.

## 3. Experiments

The proposed method was tested with in vitro acquisitions and preliminary experiments on volunteers. In vitro tests were aimed at evaluating the ability of the method to automatically locate the vessel and perform a correct flow investigation, independently of the probe positioning. Exams on volunteers were aimed at verifying the feasibility of the method in as-real conditions.

### 3.1. In Vitro Experiments Set-Up

**Figure 3:**
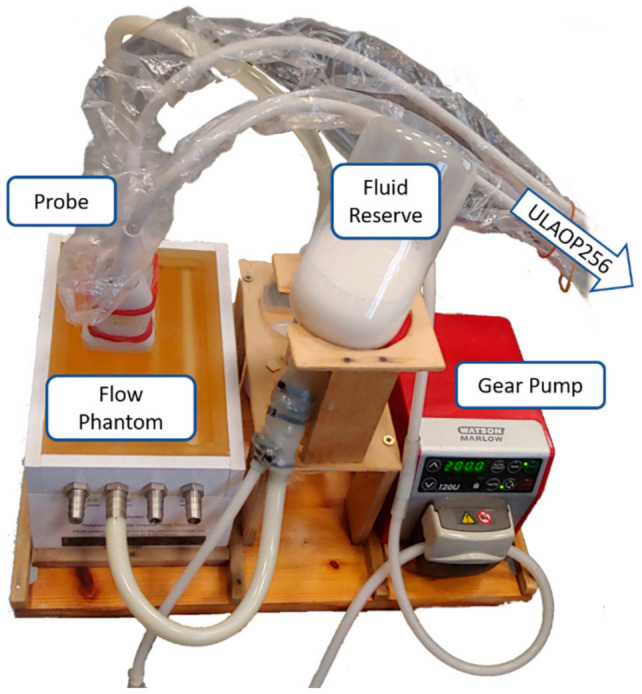
Experimental set-up. A gear pump (right) moved the blood mimicking fluid through a channel (4 or 6 mm diameter) in a wall-less phantom (left). The probe, connected to ULA-OP 256 scanner, was placed transversally on top of the phantom.

The set-up employed for the in vitro experiments is reported in Figure 3. ULA-OP 256 was connected to the probe composed by the double 128-array described in Section 2.1. The probe was located on top of the flow phantom 524 (CIRS, Norfolk, VA, USA), in a transversal position with respect to the channel under investigation. The phantom includes 2, 4, 6, and 8 mm diameter wall-less channels. In the experiments, the 4 and 6 mm diameter pipes were employed, while the others were excluded because the phantom border was visible in the B-mode image. A programmable gear pump (Watson Marlow, Wilmington, MA, USA) pushed a blood mimicking fluid at a steady velocity in the channel being investigated.

In every experiment, the operator placed the probe above the flow channel, then triggered the start of the automatic measurement. The scanner ran the operations as described in Section 2.3 and produced the velocity measurement. The scanner was also set to save the B-mode images employed for the automatic vessel localization, and the coordinates of the detected positions, which were employed in the performance evaluation of the method.

**Figure 4:**
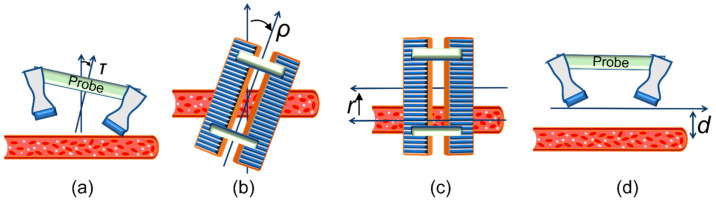
Probe positions tested in in vitro experiments: (**a**): tilt with −15° ≤ τ ≤ +15°; (**b**): rotation with 0 ≤ ρ ≤ 20°, (**c**): lateral translations with random r values; (**d**): depth with 19 ≤ d ≤ 30 mm.

Several sequences of experiments were carried out. In the first sequence, the probe was placed in transversal position with respect to the channel, with the channel crossing at about the center of the ROI. From the central position, where the probe was perpendicular with respect to the channel (and thus the Doppler angles with the 2 arrays were 90°− θ/2 = 75° and 90° + θ/2 = 105°), the probe was tilted in 9 different positions, in the range from −15° to +15° (see Figure 4a). An external support was used for tilting the probe in the desired position. In the second sequence of experiments, the probe was positioned as it was in the first series, but this time the probe was rotated around the depth axis in 6 positions in the range from 0° to 20° (see Figure 4b). An external support was used for rotating the probe in the desired position. In the third series of experiments, the operator, without the help of the ULA-OP 256 display, positioned the probe over the channel being investigated. The operator placed the probe at tilt τ = 0° and rotation ρ = 0° but without the help of external supports. The operator purposely laterally translated the probe in random positions so that the channel crossed the ROI at different lateral positions (see Figure 4c). In the last series of experiments, the operator placed the probe at tilt τ = 0°, rotation ρ = 0°, and r = 0 without the help of the B-mode display. A support was used to change the distance between probe and channel center in the range 19–30 mm.

Table 3 summarizes the characteristics of the in vitro experiments. A total of 54 experiments were carried out for the 2 tested channel sizes. The reference velocity was 34 and 18 cm/s for the 4 and 6 mm diameter pipe, respectively.

### 3.2. Experiments on Volunteers

The method was tested on the common carotid artery (CCA) of 3 healthy volunteers. The volunteer under test was seated and relaxed for 5 min in a comfortable position. The operator, without the help of the B-mode, positioned the probe on her/his neck so that the CCA crossed the ROI transversally. The operator placed the probe roughly at τ = 0° of tilt and ρ = 0° of rotation but without considering the positioning as particularly critical. The operator warned the volunteer not to move in the next few seconds and started the automatic measurement procedure.

Once the procedure was completed, the operator removed the probe, paused about 30 s, and repeated the operations for a new measurement. Three to five measurements were performed for each series on each volunteer. Each sequence lasted no more than 10 min, so that the measurements of the sequence were considered to share the same nominal blood velocity. The sequences were acquired on different volunteers, or on the same volunteers on different days and/or carotid sides. We performed, in total, 6 sequences on 3 different volunteers, for a total of 30 measurements.

### 3.3. Data Analysis

#### CCA Segmentation

The B-mode images saved during all the experiments, both in vitro or on volunteers, were processed off-line by an expert operator. The operator manually placed the investigation line as he would have with a real Doppler measurement. Then, he located the position of the sample volume in the center of the lumen along the investigation line. The position of sample volume was considered as the reference to be compared to the position of the lumen center located by the scanner as the result of the automatic procedure. The error of the automatic procedure in the location of the lumen center was evaluated, for each experiment *i*, by the geometrical distance of the automatically located and reference center positions

(3) $$De_{i}=\frac{1}{N}\;\underset{i}{\sum }\sqrt{{\left(C_{Mxi}-C_{Rxi}\right)}^{2}+{\left(C_{Myi}-C_{Ryi}\right)}^{2}}$$

In Equation (3) ($C_{Mxi}$,$\;C_{Myi}$) and ($C_{Rxi}$,$\;C_{Ryi}$) are the lateral and depth coordinates of the measured and reference centers of the carotid lumen in the experiment *i*. In cases where the distance error was higher than 1 mm, the measurement was considered unsuccessful and discarded. The total error was summarized through the mean and the root mean square value

(4) $$ELP_{T}=\sqrt{\frac{1}{N}\overset{N}{\underset{i=1}{\sum }}{\left(De_{i}\right)}^{2}}$$

**Figure 5:**
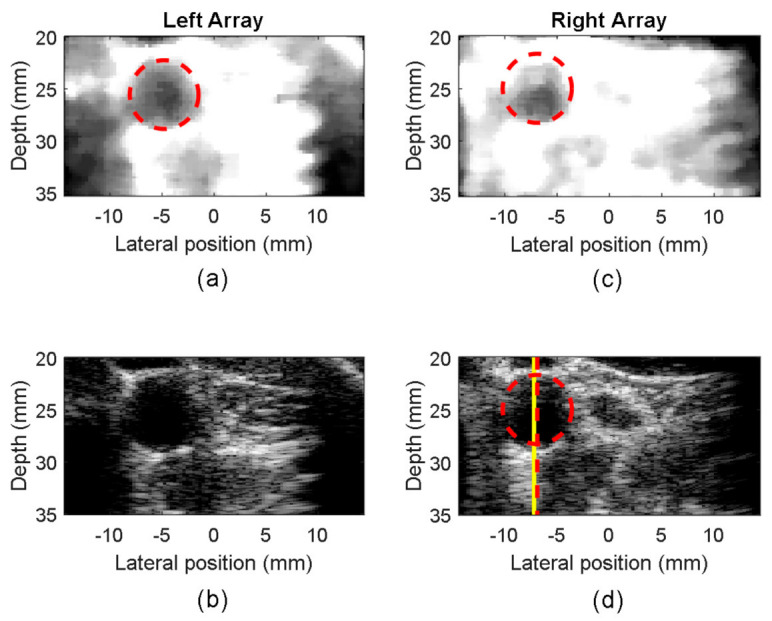
Pre-processed (**a**,**c**) and raw B-Mode (**b**,**d**) images acquired from the left (**a**,**b**) and right (**c**,**d**) arrays on a volunteer. The automatic carotid segmentation (red dashed circles), the automatic Doppler lines (vertical red dashed lines) and the reference Doppler lines (yellow vertical lines) are superimposed on the images.

Figure 5 shows, for example, the standard B-mode images (b,d) and the pre-processed images (a,c) obtained from the left (a,b) and right (c,d) arrays during the test running on one of the volunteers.

The CCA section segmented by the automatic procedure is highlighted by the red dashed circles. The center of the lumen was detected in (−4.8, 20.2 mm) and (−6.9, 20.2 mm) from the left and right arrays, respectively. The red dashed vertical lines in the B-mode images show the position of the Doppler lines automatically detected. These data should be compared to the reference Doppler line placed by the expert operator in the positions highlighted by the yellow lines. In this example, the positioning error was 0.4 and 0.3 mm for the left and right images, respectively.

For each acquisition, the angle-corrected flow velocity was obtained as described in Section 2.5. The accuracy of the measurements obtained for in vitro experiments was evaluated by comparing the velocity $V_{Mi}$, measured in experiment *i*, to the reference velocity $V_{R}$

(5) $$Err_{i}{}_{\%}=\frac{V_{Mi}-V_{R}}{V_{R}}\;\cdot 100.$$

**Figure 6:**
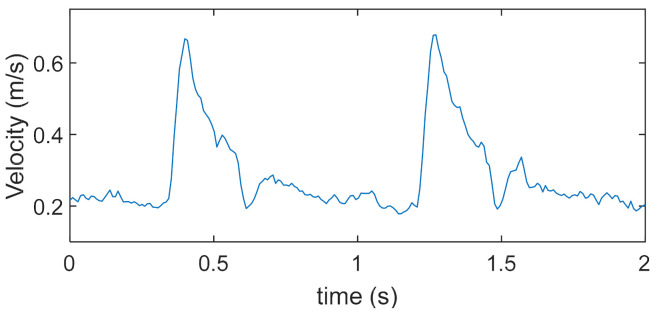
CCA blood velocity measured in one of the volunteers by the proposed method. The 2 s acquisition captured 2 systolic peaks, whose maximum was 0.67 m/s.

The variability of the measured velocity was evaluated among experiments of the same sequence. For in in vitro tests, experiments of the 4 series listed in Table 3 were matched; for the measurement performed on the CCA of the volunteers, the measurements performed in the same session were compared. The variability was then expressed through the variability coefficient, reported in percentage

(6) $$CV_{\%}=\frac{\mathrm{std}\left(V_{Mi}\right)}{V_{M}}\;\cdot 100$$

where ‘std’ is the standard deviation, $V_{Mi}$ and $V_{M}$ are the single velocity measurement and the mean among the sequence, respectively. Figure 6 reports, for example, the blood velocity trend measured in one of the volunteers. The 2 s acquisition captured 2 systolic peaks, whose maximum was 0.67 m/s. This value is the output of the measurement.

## 4. Results

### 4.1. In Vitro Channel Segmentation

The accuracy of the automatic segmentation algorithm in detecting the vessel center in the in vitro experiment is reported in Table 4. Results were elaborated separately for the channels with 4 and 6 mm diameter, and for the series where the tilt, the rotation, the lateral position, and the depth were varied (see Table 3). On the other hand, the data elaborated from the right and left B-mode sequences were merged together and not differentiated. In these experiments, the maximum error was less than 0.7 mm, with an r.m.s. value between 0.14 and 0.43 mm. In a single test, the error was 1.16 mm, and the acquisition was discarded. The number of discarded acquisitions was one out of fifty-four, thus, less than 2%. Table 4 shows that the accuracy did not depend on small rotations and tilt angles that the probe can have with respect to the nominal position. This is an important result since, from an angled point of view, the shape of the vessel lumen becomes slightly elliptical instead of circular. Experiments confirm that the performance of the automatic segmentation, which nominally detects circles, also tolerates this condition well.

### 4.2. CCA Segmentation on Volunteers

Table 5 reports the accuracy of the carotid segmentation measured on volunteers. The measured error is higher than in the in vitro experiments. The higher error is reasonably due to the higher variability of the echogenicity and shape of the real carotid wall, with respect to the uniform appearance of a phantom channel. Nevertheless, the error is in the order of 0.6 mm with a peak of 0.9 mm. Two acquisitions were discarded because the error was over the threshold.

### 4.3. In Vitro Accuracy and Repeatability

The accuracy and repeatability of the in vitro velocity measurements were evaluated by Equations (5) and (6), respectively, as described in the previous section. The results, differentiated for the diameter of the two channels and the four series of experiments, are listed in Table 6. The columns report the velocity, calculated as the mean of the peak velocities obtained in each experiment of the series (Mean Peak), the relative error ($Err_{i}{}_{\%}$), and the coefficient of variation ($CV_{\%}$). In two of the experiments with the 4 mm pipe that were discarded, the velocity was not correctly detected; in the 6 mm pipe all measurements succeeded. The error was averaged among measurements of the same series. This ranges between −5.3% and +8.3%, with a mean value of 3.2%. The $CV_{\%}$ ranges between 2.2% and 10.6%, and its mean value is 4.5%. The experiments show that the accuracy and repeatability of the velocity measurement do not depend significantly on the position of the probe, and the method is robust with respect to small probe translations, inclinations, rotations, and depth variations.

### 4.4. Repeatability on Volunteers

The results regarding the repeatability of the velocity measurements performed on volunteers are reported in Table 7. The table reports the mean systolic peak (Mean Peak), the standard deviation (std), and the coefficient of variation ($CV_{\%}$). The statistics are elaborated on the population of each sequence of acquisitions, as detailed in the previous sections. The measured systolic peaks range between 63 and 81 cm/s, which are the physiological values [22] expected in presumably healthy volunteers. The variability of the measurements is comparable to what was calculated for the in vitro experiments and, in some cases, even lower. The blood velocity was correctly detected in all of the measurements with the exception of two tests, which were discarded.

## 5. Discussion

The proposed method is intended to foster the development of small and low-cost tools for the simplified and even self-made measurement of the CCA blood systolic velocity, with the prospect of extending the advantages of the ultrasound medical technology to a wider community [4,5].

This target imposes several constraints and limitations on the probe geometry, the choice of the method, the investigation site, the measurement configuration, and the signal processing; some of these are briefly discussed below and summarized in Table 8 and Table 9.

The typical clinical CCA exam is carried out in a longitudinal position [1], but only an expert operator can correctly align a linear array to the carotid in a longitudinal view. A 2D matrix [8] would allow a wider view where a non-expert would also have a good chance to locate the vessel. However, the use of a 2D matrix is too complex for the proposed target. On the other hand, the CCA can easily be detected in the large field of view of a linear array placed in the transversal position, even without the help of a B-mode display. However, in this view, the Doppler angle correction [1] is not possible with a single array. The proposed geometry, based on two angled arrays, was chosen here because it features both a large field of view and the possibility of automatic angle correction.

Unfortunately, this choice comes at a cost. The inclination of the two linear arrays is fixed and, thus, the beams cross at a fixed depth. Because of the physiological variability of the carotid depth [22], the beams cross point will not coincide exactly with the vessel center in all of the examined people and, therefore, a measurement error can be produced. However, the reported experiments show no particular performance decrease (see Table 6) in the IV series (where vessel depth is changed) with respect to the series I–III (where the vessel center is aligned with the beam cross point). Since there is a relatively low inter-angle of 30°, the beams run close to each other for a relatively large tract of space near the crossing point and, thus, at the same depth they intercept flow lines with a similar velocity. With the vessel being almost perpendicular to the beams mid-axis, and with the flow being almost laminar, this further reinforces the aforementioned interpretation of the experimental results.

A complete clinical exam of the carotid artery is not limited to CCA, but often also includes the measurement of other parameters such as, for example, the blood velocity in the internal and/or external branches [2], the wall movement [28,29], and the intima–media thickness [30]. Although automatic segmentation algorithms are available [31], the image acquisition cannot be automatized easily, and the supervision of an expert is still needed for these measurements. The employed set-up would allow the estimation of the volume flow by the integration of the velocity profile over the diameter [32] as well, but we preferred to limit the output to a single parameter so as not to compromise the simplicity of its use. Finally, the CCA blood velocity peak is also of high medical interest when taken alone [33].

Clinical echographs measure the blood velocity from the envelop of the spectrogram [1]. This measurement is sensitive to the spectral broadening [34,35], which in turn is affected by several geometrical and configuration parameters, such as the angle [36]. In this study, we preferred employing the spectral frequency centroid, which, joined to the dual-beam vector technique, was already proven to be a better estimator of blood peak velocity with respect to spectral envelop [37,38]. However, this estimator requires a small sample volume: in the presented experiments we used three cycles @ 7.5 MHz and a 1 MHz low pass filter after demodulation, for an axial extension of the sample volume of about 0.6 mm.

The method employs the acquisition parameters listed in Table 2; in particular, we used a fixed PRF of 8 + 8 kHz, i.e., 8 kHz for each beam. This allows reaching a maximum depth of 9 cm, well beyond the typical carotid depth [22]. Moreover, the carotid is roughly parallel to the skin so the Doppler angle for each beam is about 75° (i.e., 90°–30°/2), where 30° is the beam inter-angle. Thus, the maximum detectable velocity, using the full PRF range, is around 3 m/s, which is suitable for the application.

The large field of view of the array (29 mm) makes it quite easy to intercept the carotid vessel in the neck tract, even without the help of the B-mode display [39]. However, the non-expert operator could erroneously intercept the carotid bifurcation or the jugular vein instead of the CCA. Although the algorithm cannot help in this part of the positioning, finding the right vessel region is relatively easy. In addition, the non-expert operator is not expected to achieve a perfect probe positioning: the experiments show that the method easily tolerates errors of translation, tilt, and rotation of the probe. Moreover, the method includes some basic techniques to exclude measurements that are obviously incorrect. For example, if the algorithm erroneously locks on the jugular vein, the velocity measurement will feature a wrong velocity direction, and the measurement will be discarded. Thanks to these checks, the operator can easily and quickly repeat measurements that have failed due to an erroneous probe position, patient movement, or for other reasons.

In this study, precision of the method was evaluated both in vitro and on volunteers, while the accuracy was evaluated in vitro (see Table 6 and Table 7). The evaluation of the accuracy in volunteers/patients needs a statistical study on a large number of subjects that goes beyond the aim of this pilot study.

The proposed implementation is based on a probe composed of two commercial linear arrays joined by a holder. The result is a cumbersome, heavy, and uncomfortable probe. An integrated probe based on PMUT technology is currently under development [40]; it is expected to significantly improve the usability of the method.

The proposed method, when associated with wearable devices [41,42], can also be exploited for long-term monitoring of the peak CCA velocity in patients out of the clinical environment, possibly speeding up their dismissal from hospitals.

**Table 8:** Method limitations.

Limitations	Workarounds/Notes
Analysis in transversal position only	none
Beams cross at a fixed depth	Experiments show that the method works in a relatively wide depth-range
Measurement of peak velocity only	Peak velocity has medical interest [33]
Possibility to erroneously intercept the jugular vein	Measurement automatically discarded
Cumbersome probe	Replaced by an integrated PMUT array

**Table 9:** Method strengths.

Strengths	Notes
Completely automatic	The method can be used by a non-expert user, or even by the patient alone
Automatic angle correction	The method compensates for Doppler angle
Large field of view	The carotid is located even without the B-mode display
Automatic check for measurement correctness	Wrong measurements are automatically discarded
Low cost	It can be implemented in a small and economic device

## 6. Conclusions

A method for the automatic measurement of the CCA blood velocity peak was presented. The method is based on the automatic segmentation of the CCA in the B-mode image, followed by the angle-corrected velocity measurement. Although more tests are necessary, it is hoped that this method can contribute to the improvement of the quality of life in all of the places where the access to a full medical service is limited, including modern cities.

## Figures and Tables

**Table 1:** Probe details.

Probe	L12-5D
Probes Type	Linear
Elements	128
Central frequency	7.5 MHz
Bandwidth (−6 dB)	80%
Element Pitch	300 μm
Bandwidth	6–11 MHz
Elevation focus	20 mm

**Table 2:** Transmission–Reception strategy.

B-Mode Image	
Lines	96
Frequency	7.5 MHz
TX focus	20 mm
RX focus	Dynamic F# = 3
**Doppler Image**	
Packet Size	128
TX/RX Aperture	16 elements (4.8 mm)
TX Azimuthal Focus	20 mm
Transmission	3 sinusoidal cycles @ 7.5 MHz
PRF	8 + 8 kHz
Reception Focus	Dynamic focusing F# = 3
Clutter filter	100 Hz

**Table 3:** In vitro experiments.

Series	Tilt τ (°)	Rot. ρ (°)	Transl. r (mm)	Depth d (mm)
I	−15, −10, −6, 0, +6, +10, +15	0	0	23
II	0	0, 3, 6, 12, 16, 20	0	23
III	~0	~0	random	23
IV	~0	~0	0	19–30

**Table 4:** In vitro segmentation error.

Series	Min/Max (mm)	Mean (mm)	r.m.s. (mm)	Discarded
4 mm channel				
I (Tilt)	0.03/0.60	0.19	0.23	0
II (Rotation)	0.03/0.29	0.11	0.14	0
III (Translation)	0.04/0.64	0.24	0.27	1
IV (Depth)	0.02/0.60	0.21	0.30	0
6 mm channel				
I (Tilt)	0.08/0.53	0.23	0.26	0
II (Rotation)	0.09/0.52	0.26	0.29	0
III (Translation)	0.03/0.67	0.26	0.31	0
IV (Depth)	0.30/060	0.41	0.43	0

**Table 5:** Segmentation error on volunteers.

Series	Min/Max (mm)	Mean (mm)	r.m.s. (mm)	Discarded
I	0.06/0.83	0.36	0.42	0
II	0.19/0.59	0.40	0.42	0
III	0.06/0.55	0.24	0.29	0
IV	0.20/0.80	0.56	0.62	0
V	0.01/0.62	0.57	0.44	1
VI	0.11/0.90	0.45	0.50	1

**Table 6:** Velocity error and repeatability.

Series	Mean Peak (cm/s)	$Err_{i}{}_{\%}$ (%)	$CV_{\%}$ (%)	Discarded
4 mm channel				
I (Tilt)	29.7	−5.3	10.6	0
II (Rotation)	32.9	+2.1	5.2	1
III (Translation)	35.5	+4.4	4.2	1
IV (Depth)	36.0	+5.9	5.5	0
6 mm channel				
I (Tilt)	19.5	+8.3	2.8	0
II (Rotation)	18.5	+2.8	2.9	0
III (Translation)	18.8	+4.4	2.2	0
IV (Depth)	18.7	+3.7	3.0	0

**Table 7:** Velocity repeatability on volunteers.

Series	Mean Peak (cm/s)	Std (cm/s)	$CV_{\%}$ (%)	Discarded
I	69.2	8.4	12.1	0
II	68.5	5.2	7.7	1
III	63.5	4.6	7.3	1
IV	74.0	5.3	7.1	0
V	57.0	3.4	6.1	0
VI	81.0	8.1	10.1	0

## Data Availability

Raw experimental data are available from authors.
